# Influence of Fermentation and Drying Materials on the Contamination of Cocoa Beans by Ochratoxin A

**DOI:** 10.3390/toxins5122310

**Published:** 2013-11-28

**Authors:** Sébastien Djédjé Dano, Pierre Manda, Ardjourma Dembélé, Ange Marie-Joseph Kouassi Abla, Joel Henri Bibaud, Julien Zroh Gouet, Charles Bruno Ze Maria Sika

**Affiliations:** 1 Laboratory of Toxicology, UFR Pharmaceutical and Biological Sciences, University Félix Houphouet Boigny, B.P.V. 34 Abidjan, Côte d’Ivoire; E-Mails: sebastien.dano@gmail.com (S.D.D.); majoa24@hotmail.fr (A.M.-J.K.A.); drhenjel@ymail.com (J.H.B.); juliengouet@yahoo.fr (J.Z.G.); zsika@yahoo.fr (C.B.Z.M.S.); 2 Laboratory of Agrochemistry and Ecotoxicology, National Laboratory for Agriculture Development, 04 B.P. 612 Abidjan, Côte d’Ivoire; E-Mail: ardjouma@yahoo.fr

**Keywords:** ochratoxin A, cocoa, drying platforms, fermentation materials, Côte d’Ivoire

## Abstract

Ochratoxin A (OTA) is a mycotoxin produced mainly by species of *Aspergillus* and *Penicillium*. Contamination of food with OTA is a major consumer health hazard. In Côte d’Ivoire, preventing OTA contamination has been the subject of extensive study. The current study was conducted to evaluate the influence of fermentation and drying materials on the OTA content in cocoa. For each test, 7000 intact cocoa pods were collected, split open to remove the beans, fermented using 1 of 3 different materials, sun-dried on 1 of 3 different platform types and stored for 30 days. A total of 22 samples were collected at each stage of post-harvesting operations. The OTA content in the extracted samples was then quantified by high-performance liquid chromatography. OTA was detected in beans at all stages of post-harvesting operations at varying levels: pod-opening (0.025 ± 0.02 mg/kg), fermentation (0.275 ± 0.2 mg/kg), drying (0.569 ± 0.015 mg/kg), and storage (0.558 ± 0.04 mg/kg). No significant relationships between the detected OTA level and the materials used in the fermentation and drying of cocoa were observed.

## 1. Introduction

Ochratoxin A (OTA) is a mycotoxin produced by fungi, belonging mainly to species of the genera *Aspergillus* and *Penicillium* [1]. OTA contaminates myriad agricultural products, such as cereals, dried fruits, coffee, and cocoa [2,3,4], as well as some manufactured agricultural products, including chocolate, wine, beer, and bread [5,6,7]. The consumption of these OTA-contaminated foods may lead to human and animal diseases. Indeed, OTA is involved in Balkan endemic nephropathy [8] and is genotoxic, immunotoxic, and carcinogenic [8,9,10,11,12,13]. Previous animal studies have demonstrated the genotoxic, carcinogenic, and immunotoxic effects of OTA *in vivo*. Based on available evidence, OTA is classified as a Group 2B possible human carcinogen by the International Agency for Research on Cancer (IARC).

The health risks associated with the consumption of OTA-contaminated foods necessitate drastic measures to protect worldwide consumer health. Accordingly, in the European market, food products are subject to maximum allowable threshold concentrations of OTA. The EU 1881/2006 regulation sets maximum levels of OTA in foodstuffs: 5 µg/kg in cereals, 3 µg/kg in cereal-processed products, 5 µg/kg in raisins, 5 µg/kg roasted coffee and ground coffee, 10 µg/kg in instant coffee, and 2 µg/kg in wine and spices [14].

The European Food Safety Authority (EFSA) established an acceptable weekly intake of OTA of 120 ng/kg body weight in 2006 [9]. The European Commission recently declined to set a maximum acceptable level for OTA in cocoa and cocoa-derived products because these products do not contribute significantly to OTA exposure and high levels of OTA are rarely detected in these commodities [15].

However, to minimize the OTA contamination of cocoa and ensure high-quality cocoa, cocoa-producing countries should develop post-harvest treatment guidelines. Indeed, analysis of cocoa bean samples from different countries of origin has revealed the presence of OTA at variable quantities for samples collected from different regions or even within the same region [7,16,17,18,19,20,21,22,23,24].

Critical points of entry for OTA contamination and factors favoring the production of OTA in cocoa have been investigated previously. According to Bastide *et* *al*. [25], the OTA levels in cocoa vary from one cocoa season to another and are also dependent on the physical condition of the cocoa pods. These authors reported that pods damaged by insects, mutilated pods, rotting pods, and mummified pods exhibited greater OTA contamination compared to healthy pods.

The contamination of cocoa beans with OTA occurs mostly during post-harvest operations: harvesting, pod-breaking, fermentation, drying, storage, and transportation. These operations require the use of various rudimentary materials and utensils that are sometimes reused for several cocoa seasons. For example, in Côte d’Ivoire, cocoa fermentation is performed on the following materials: banana leaves, wooden boxes or on black tarpaulins. Similarly, sun-drying is performed on different platforms, including rack tables (seco), concrete floors, and black tarpaulins.

The main objective of this study was to evaluate the influence of the materials used in post-harvest operations, including fermentation, drying, and storage, on the presence of OTA in cocoa beans. The results of this study will be used by the Committee on a Strategy for the Prevention of Contamination of OTA in Coffee and Cocoa in Côte d’Ivoire.

## 2. Results and Discussion

### 2.1. Analytical Method

The method employed herein was previously validated by Manda *et* *al*. (2009). The analytical conditions are summarized below. OTA extraction was performed under alkaline conditions, as previously described [17,26]. This method ensured excellent recovery (95.2% ± 0.5%). The percent recovery was very consistent, and RSDs were lower than 3%, demonstrating the precision of the analytical procedure. Thus, this method is valid according to Directive 2002/26/CE, which indicates that recovery quantities of <1 µg/kg are acceptable within a range of 50%–120% total recovery. All data were corrected according to the overall recovery (95.2% ± 0.5%). The coefficients of variation for repeatability and reproducibility were 0.70% and 2.2%, respectively. The limit of detection (LOD) and limit of quantification (LOQ) were 0.05 and 0.2 µg/kg, respectively. When constructing dose-response curves for OTA analysis, solutions containing 0.05, 0.2, 0.5, 1, or 2 µg/kg were used as controls, and the coefficient of linearity (*R*^2^) was 0.9995. These results demonstrate the reliability of the method used to determine OTA levels in cocoa bean samples.

### 2.2. Collection of Samples

Samples were collected during two cocoa seasons. Sample collection was conducted in three distinct cocoa-producing regions of Côte d’Ivoire (east, central, and southwest). Sample preparation, including cocoa harvesting, pod-breaking, fermentation, drying, and storage, was repeated 17 times between 2008 (4 times) and 2009 (13 times). A total of 374 samples were collected from three study sites to evaluate the effects of pod-opening (17 samples), fermentation (51 samples), drying (153 samples), and storage (153 samples) on OTA content.

### 2.3. OTA Content in Different Cocoa Samples

The data are presented as the average level of OTA contamination for each sample group for different post-harvest operations.

### 2.4. Pod-Opening

The OTA content detected in different samples collected during the pod-breaking stage is reported in Table 1.

The OTA content ranged from <LD (limit of detection) to 0.1 µg/kg (Table 1). No significant differences were observed between the samples collected from the 3 locations. The overall average OTA content was 0.038 ± 0.02 µg/kg. The OTA levels detected in positive samples were very low, all below both the detection limit (0.05 µg/kg) and the limit of quantification (0.2 µg/kg). Beans from intact cocoa pods had minor OTA contamination.

According to Schwan and Wheals [27], the interior of an intact cocoa pod is sterile. Microorganisms may enter beans during fermentation via exposure to contaminated surfaces during pod opening, such as the hands of workers and/or the equipment used, including machetes and other fermentation materials. Our results corroborate those of Bastide *et al*. [25]. Mounjouenpou *et* *al*. [28] also reported similar OTA concentrations, on the order of 0.03 ± 0.00 µg/kg and 0.08 ± 0.01 µg/kg, in samples from intact cocoa pods stored 10 days before pod-opening. The strict sample collection criteria are likely responsible for the low OTA concentrations observed at this stage of pod-opening. All pods that had visible defects on the outer cortex, including signs of parasitic infection, black pod, rotten pod, and damaged pods, were excluded from the study.

**Table 1:** Ochratoxin A (OTA) contamination level following pod-opening.

		Range		Area		
	Average	Min	Max	Abengourou ( *n* = 4)	Gagnoa ( *n* = 4)	San Pedro ( *n* = 9)
OTA level (µg/kg)	0.038 ± 0.025 (12/17)	<LD	0.1	0.056 ± 0.04 ^a^	0.052 ± 0.023 ^a^	0.025 ± 0.026 ^a^

^a^ Averages denoted by the same letter are not different.

### 2.5. Fermentation

After pod-opening, the cocoa beans extracted from the pods were fermented using 1 of 3 different materials: banana leaves, wooden boxes, or black tarpaulins. The mean sample contaminations for the different fermentation materials are reported in Table 2. No significant difference was observed between the type of fermentation material used (*p* = 0.436).

**Table 2:** Average contamination levels as a function of the materials used in fermentation.

		Range		Fermentation material		
	Average	Min	Max	Tarpaulins ( *n* = 17)	Banana leaves ( *n* = 17)	Boxes( *n* = 17)
OTA level (µg/kg)	0.275 ± 0.26 (29/51)	<LD	2.1	0.366 ± 0.39 ^a^	0.23 ± 0.15 ^a^	0.228 ± 0.22 ^a^

^a^ Means denoted by the same letter are not different (*p* = 0.436).

All of the measured OTA concentrations approached the LOQ. Cocoa bean fermentation is the first step of the cocoa bean transformation process. Indeed, many parameters, including a variety of microorganisms, temperature, and pH, are involved in this stage of cocoa bean processing. Changes to the chemical composition of the beans during this processing culminate in not only significant changes in the organoleptic characteristics of cocoa but also the proliferation of microorganisms, including those likely to produce OTA.

Mounjouenpou *et* *al*. [29] reported a large increase in filamentous fungal species after fermentation, possibly due to of the presence of sweet mucilage.

Fermentation of beans from intact pods may prohibit OTA contamination or limit its presence to trace quantities from natural materials.

### 2.6. Drying

Three different types of drying platforms were also used during the post-harvest treatment. Each batch of fermented beans obtained from the previous fermentation step was divided into three sub-lots and dried separately on 1 of the 3 drying platforms, which included rack tables, a concrete floor, or black tarpaulins. The average OTA contamination level was 0.569 µg/kg (Table 3). The type of drying platform did not affect the OTA concentrations, which were equivalent for samples from the rack table (0.459 ± 0.04 µg/kg), tarpaulin (0.665 ± 0.02 µg/kg), and concrete floor (0.584 ± 0.02 µg/kg). An analysis of variance did not reveal any significant differences among the samples generated using the three materials studied (*p* = 0.834). Similar results were also reported by Karine *et al*. in 2001 [30].

**Table 3:** Levels of OTA contamination as a function of the drying platform used.

		Range		Drying platform		
	Average	Min	Max	Rack	Black tarpaulin	Cement floor
OTA level (µg/kg)	0.569 ± 0.3	<LD	13.4	0.459 ± 0.04 ^a^	0.665 ± 0.023 ^a^	0.584 ± 0.026 ^a^

^a^ Means denoted by the same letter are not different (*p* = 0.834).

### 2.7. Storage

Samples were maintained in the cocoa cooperatives’ urban stores after drying and were then collected for OTA sampling 30 days later. The OTA concentrations observed following the storage period were identical to the values obtained following the drying process. The average OTA concentration, 0.558 ± 0.04 µg/kg (Table 4), was likely due to appropriately maintained storage conditions, with bags on pallets and good ventilation. The OTA content in the beans after 30 days of storage was not influenced by the storage materials (jute bags) or by the fermentation and drying materials. Our results differ from those of Mounjouenpou *et al*. [28], who reported increased OTA levels after 2 and 4 months of storage. Our results are in agreement with those of Wood *et al*. [31] for the storage of cocoa beans in tropical countries and support the directive that storage should not exceed 2 to 3 months. Beyond three months, mold growth often develops due to the hygroscopic properties of cocoa beans. Less than 8% moisture content is recommended for proper conservation of cocoa beans.

**Table 4:** OTA contamination levels following storage as a function of drying platform type.

	Rack	Black tarpaulin	Cement floor
OTA level (µg/kg)	0.600 ± 0.025 ^a^	0.577 ± 0.032 ^a^	0.419 ± 0.29 ^a^
Average level at the end of storage (µg/kg)	0.558 ± 0.3		

^a^ Means denoted by the same letter are not different.

### 2.8. General Assessment of Contamination during Post-Harvest Operations

OTA was detected in varying quantities in beans at all stages of post-harvest operations. OTA was detected in trace quantities at the pod-opening stage (0.025 ± 0.02 µg/kg), in increased concentrations following fermentation (0.275 ± 0.2 µg/kg), and at equivalent levels following drying (0.569 ± 0.015 µg/kg) and storage (0.558 ± 0.04 µg/kg).

Post-harvest operations can be grouped into 2 phases based on the detected OTA content: pod breaking and fermentation, during which the OTA concentrations were very low, and drying and storage, during which the OTA concentrations were high. The OTA levels significantly increased during the transition from fermentation to drying. Thus, our results suggest that the drying operations, which consisted of moisture content reduction from 60% to below 8% in the fermented beans, introduced significant OTA contamination (Table 5). Our results are in accordance with those of Copetti *et al*. [22], who measured OTA at all stages of cocoa processing and observed increased contamination due to drying (<0.01 to 5.54 µg/kg) and storage (<0.01–4.64 µg/kg) stages.

**Table 5:** Assessment of OTA contamination during each phase of the post-harvest operations.

	Pod-opening	Fermentation	Drying	Storage
OTA level (µg/kg)	0.038 ± 0.02 ^a^	0.28 ± 0.26 ^a^	0.57 ± 0.3 ^b^	0.56 ± 0.3 ^b^

^a, b^ Means denoted by the same letter are not different.

To prevent excessive contamination of beans by OTA, drying must be conducted as rapidly as possible, preferably immediately after harvest and utilizing a hot air system. The bags used for bagging should be clean, dry, and placed on pallets. Similarly, the transport system must be completely clean and free of mold, insects, or contaminated equipment.

The contamination increased during the post-harvest operations as follows: pod-opening (0.038 ± 0.025 µg/kg), fermentation (0.28 ± 0.26 µg/kg), drying (0.57 ± 0.3 µg/kg), and storage (0.56 ± 0.3 µg/kg).

## 3. Experimental Section

### 3.1. Study Sites

In Côte d’Ivoire, cocoa is grown in forest zones (13 total zones). For this study, we collected our samples from three different zones: the eastern zone, Abengourou (Yakassé Feyassé); the central zone, Gagnoa (Tehiri); and the southwestern zone, San Pedro (Gabiadji). At each site, over 2500 m^2^ was cleared for material installation. The space was divided into three functional areas: a pod-opening perimeter, a fermentation zone, and a drying area comprising six rack tables, six tracks of cement (concrete floor), and a bare, muddy floor prepared with black tarpaulin.

### 3.2. Preparation of Cocoa Samples—Bean Collection

**Figure 1:**
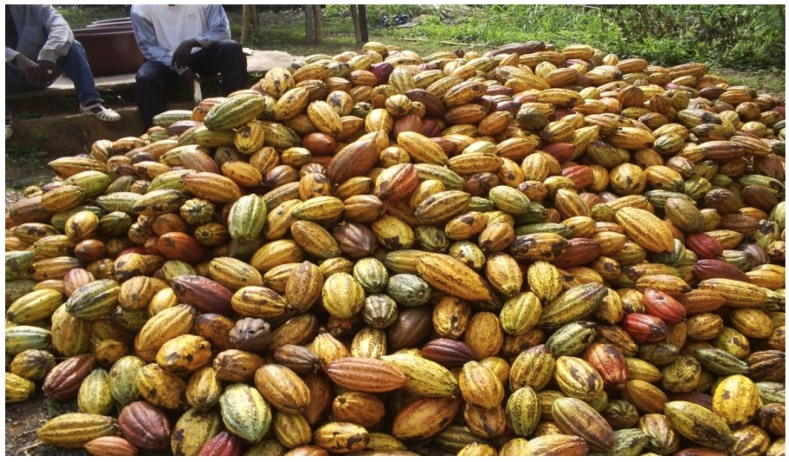
7000 intact pods assembled for one sampling event.

Samples of cocoa (*Theobroma cacao* L.) were collected during the 2008 and 2009 seasons. For each test, 7000 intact pods were collected (Figure 1). The pods were collected from different farms belonging to different farmers in the region. The selection of pods was an important factor. An intact pod is defined as a ripe pod (yellow or orange in color) that shows no defects on the outer cortex and is unopened. Black pods, rotten pods, pods attacked by insects, and pods damaged during harvesting operations were excluded. Similarly, pods containing defective beans (colored spots or the presence of mold) were also excluded. 

### 3.3. Post-Harvesting Operations

#### 3.3.1. Pod-Breaking

The harvested pods were assembled over five days. Using clubs, the 7000 cocoa pods were opened, and the beans were removed manually. A 4-kg sample of fresh beans (not fermented beans) was collected and stored in a freezer on site. The remaining beans were then divided into three lots.

#### 3.3.2. Fermentation

**Figure 2:**
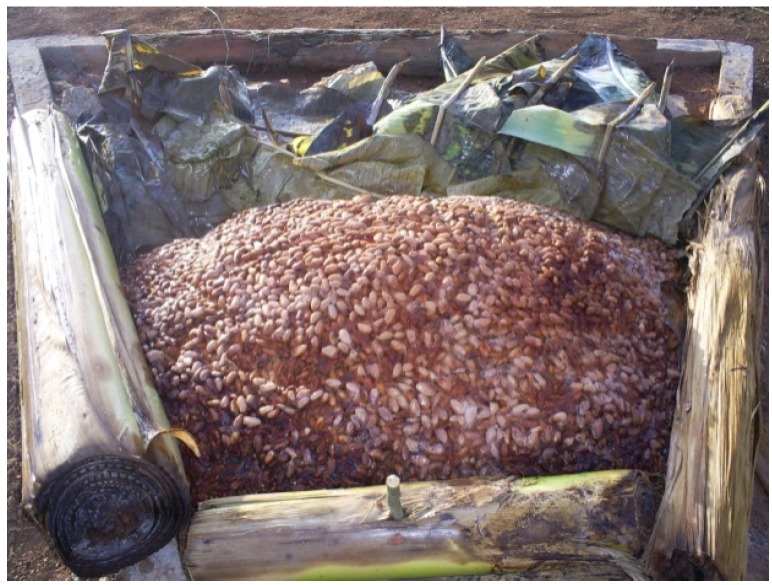
The results of fermentation on banana leaves.

**Figure 3:**
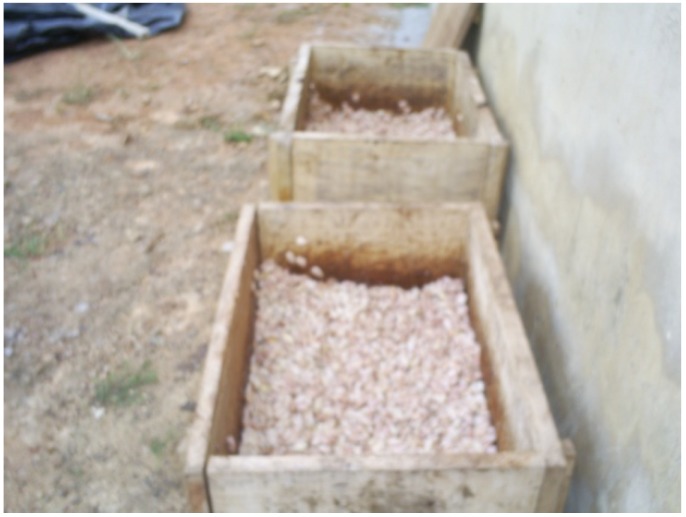
Cocoa beans in wooden boxes.

**Figure 4:**
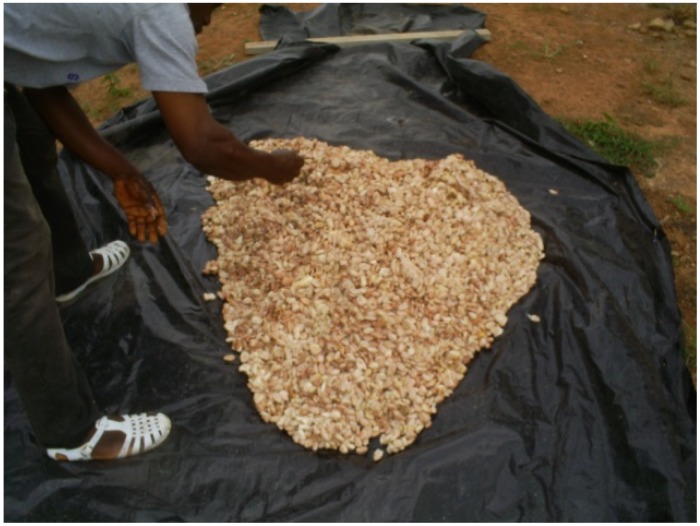
Cocoa beans on black tarpaulin.

Each of the three lots of beans from the pod-opening stage was fermented for five days on 1 of 3 different fermentation materials. These three materials were chosen for this study because they are frequently utilized by cocoa farmers in the post-harvest fermentation process. These materials included banana leaves (*Musa paradisiaca* L.), which are the traditional fermentation material commonly used by small-scale cocoa farmers. A heap of cocoa beans was piled on banana leaves spread on the ground and covered by other banana leaves (Figure 2). The second lot of fresh beans was fermented in a wooden box (60 × 60 × 60 cm) with a perforation in the bottom to facilitate pulp drainage (Figure 3). The third lot was fermented on black tarpaulin (polythene paper), which was spread on sloping ground and covered with a portion of the same black tarpaulin sheet (Figure 4). Black tarpaulin is the most commonly used cocoa fermentation material in Côte d’Ivoire. Black tarpaulin is increasingly used because of its many advantages: low cost, ease of use, and re-usability. At the end of the fermentation process, 4-kg samples were taken from each lot and kept in cold storage (−20 °C).

#### 3.3.3. Drying

**Figure 5:**
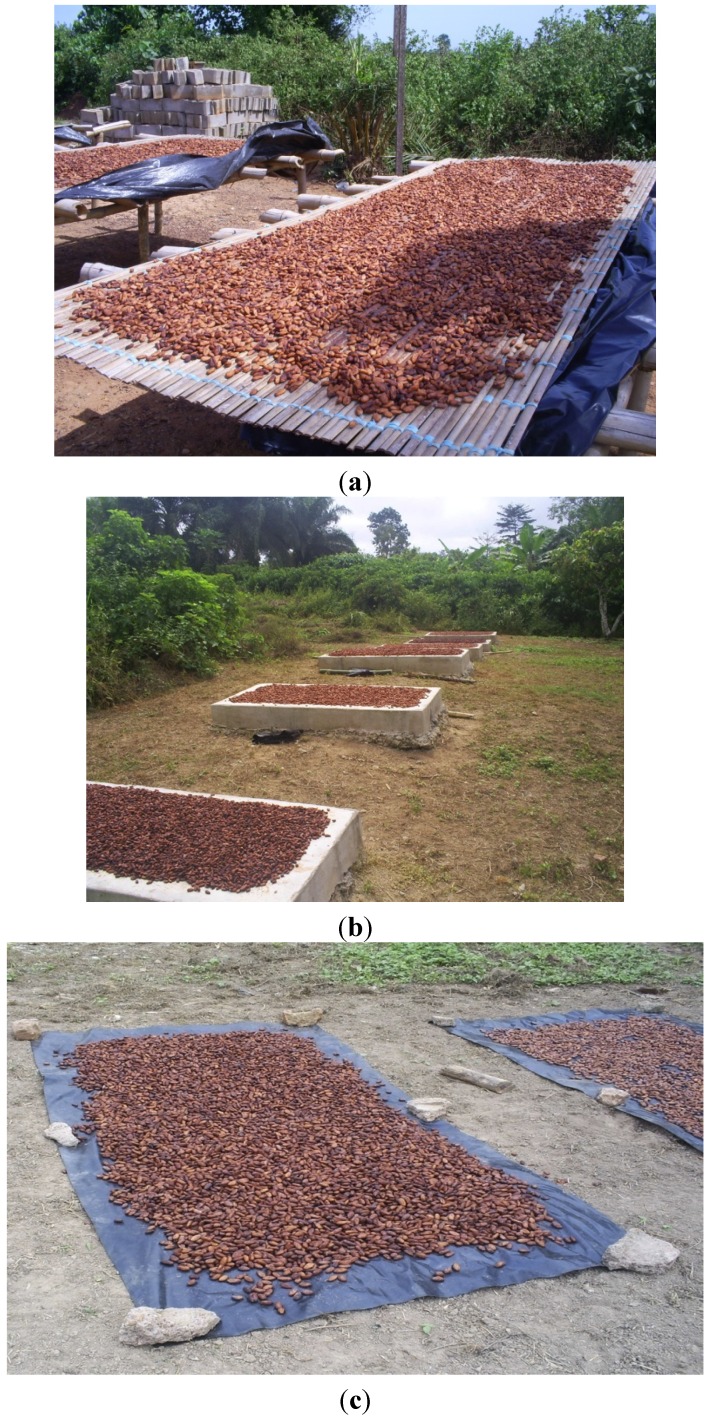
Drying platforms used in this study: drying rack table (**a**), concrete floor (**b**), and black tarpaulin (**c**).

At the end of cocoa fermentation, each lot of fermented beans from each type of material was divided into 3 sub-lots. The 3 sub-lots of fermented beans were dried in the sun on 1 of 3 types of drying platforms: a drying rack table (Figure 5a), a concrete floor (Figure 5b), or black tarpaulin (Figure 5c). At the end of the drying process, samples were collected for each experimental case. Depending on the weather conditions, drying lasted from 5 to 10 days.

#### 3.3.4. Storage

**Figure 6:**
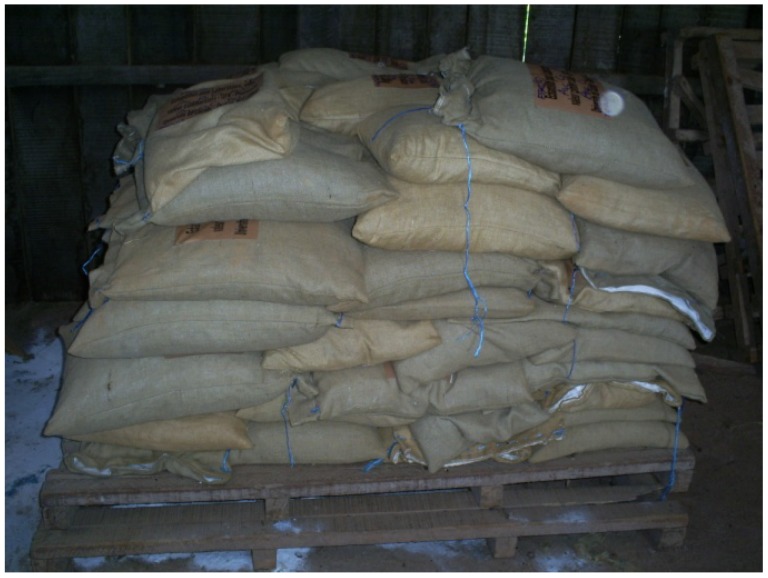
Storage of dried cocoa beans.

**Figure 7:**
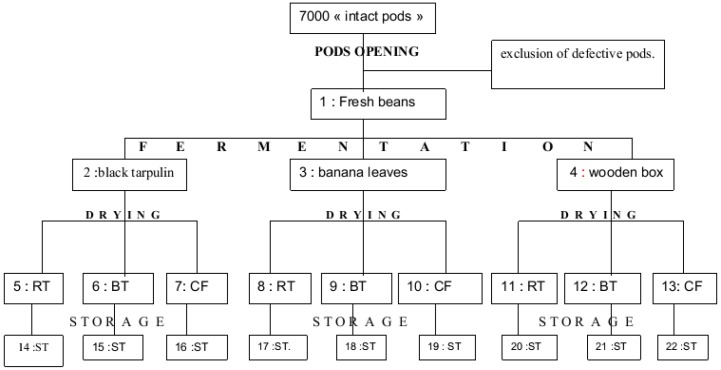
Sampling points. RT: rack table; BT: black tarpaulin; CF: concrete floor; ST: storage.

The dried cocoa beans were packed in jute bags and stored in the cooperative store in town (Figure 6). Samples were collected after 30 days of storage. Figure 7 illustrates the sampling points for each test.

### 3.4. Treatment of Samples in the Laboratory

Fresh samples (fresh beans and fermented beans) taken from the collection sites were dried in an oven at 30 °C until a moisture content of less than 8% was achieved. All dried bean samples were crushed and homogenized. A final sample of 200 g was apportioned, labeled, and stored in a cold room (−80 °C) until analysis.

### 3.5. Determination of OTA—Extraction, Detection, and Quantification of OTA in Samples

The method of determination of OTA was previously described by Manda P. *et al*. [32]. Approximately 200 g of beans were thinly ground. A 15-g sample was then placed into the bowl of a mixer (Waring blender, Torrington, CT, USA), and an aqueous solution (150 mL) (50:50, *v*/*v*) of methanol/sodium bicarbonate 3% (*m*/*v*) was added. The mixture was then stirred for 2 min. After decanting and filtering using Whatman paper (N°4), 11 ml of the filtrate was added to an equivalent volume of PBS buffer. The immunoaffinity column (R-Biopharm, Lyon, France) was pre-conditioned with 10 mL of PBS buffer at a flow rate of 3 mL/min. Then, 20 mL of the extract was loaded onto the immunoaffinity column at a flow rate of 1–2 mL/min. The OTA present in the samples was captured by the antibodies present in the agar suspension. The immunoaffinity column was washed with 20 mL of PBS buffer to remove nonspecific components. OTA was slowly eluted in 1.5 mL of a mixture of acetic acid/methanol (2:98, *v*/*v*) at a rate of 1–2 drops/s. Then, the column was washed with 1.5 mL of distilled water to obtain a final volume of 2.8 mL. After stirring, analysis was performed by high-performance liquid chromatography (HPLC).

OTA was quantified using an HPLC system consisting of a degasser (Shimadzu degaser DGU 14A Canby, OR, USA), a pump (model LC-10 ADVP, Shimadzu, Canby, OR, USA), an automatic sample injector (Shimadzu SIL-20A, Canby, OR, USA), a fluorescence detector (model RF-10AXL Shimadzu, Canby, OR, USA), and a system controller (CBM-20A, Shimadzu, Canby, OR, USA). The whole mechanism was controlled using the Lab Solution (Shimadzu, Canby, OR, USA) software.

HPLC analysis was performed in isocratic mode using fluorimetric detection at excitation and emission wavelengths of 333 and 460 nm, respectively. The mobile phase was a mixture of acetonitrile/water/glacial acetic acid (55:43:2, *v*/*v*), and the stationary phase was a C18 S5 ODS 2.5 µm (25 cm × 4.6 mm) column equipped with a pre-column. The OTA peak in the samples was identified by comparison with standards. OTA was quantified by measuring the peak area, taking into account dilution during OTA extraction and purification.

### 3.6. Statistical Analysis

Data are expressed as the mean ± SEM. The occurrence of OTA in samples was compared using a Wilcoxon matched-pair test, and statistical significance was determined by *p* < 0.05.

## 4. Conclusions

In this study, the impact of certain materials used in post-harvest operations on the OTA content of cocoa was assessed. Beans from healthy pods generally lacked OTA contamination or contained OTA in trace quantities only. We failed to observe a relationship between the observed OTA content in cocoa and the materials used during different post-harvest operations. Notably, OTA concentrations did not depend on the materials used during fermentation (banana leaves, black tarpaulin, or wooden boxes) or on the materials used during drying (rack table, black tarpaulin, or concrete floor area). The OTA concentrations were significantly higher at the end of the drying process. The materials and platforms mentioned herein did not favor OTA production in cocoa beans.
